# Elucidating the child’s perspective in health promotion: children’s experiences of child-centred health dialogue in Sweden

**DOI:** 10.1093/heapro/daaa060

**Published:** 2020-07-04

**Authors:** Mariette Derwig, Irén Tiberg, Inger Hallström

**Affiliations:** Faculty of Medicine, Department of Health Sciences, Lund University, Margaretavägen 1 B, 22240, Lund, Sweden

**Keywords:** child-centred, child health services, health literacy, health promotion, primary prevention obesity

## Abstract

Promoting young children’s health through health promotion activities is an investment for the future. In the Child Health Services in the south of Sweden a structured Child-Centred Health Dialogue (CCHD) directed to all 4-year-old children was developed using illustrations based on the most important health messages associated with the promotion of healthy lifestyle in preschool children. The aim of this study was to explore the experiences of children participating in CCHD using 21 non-participant observations during their 4-year health visit and additionally 16 individual interviews 0–7 days after their visit, conducted in the child’s home in the presence of a caregiver. *Children participated as social actors when guided to express their views based upon their own understanding* was the overall main category generated from the qualitative content analysis. The children showed that they liked to participate actively but could influence their choice to participate. They expressed their views based on their daily life and wanted to understand the meaning of the information with which they interacted. The study revealed that 4-year-old children given the opportunity to speak for themselves—elucidating the child’s perspective—interpreted the health messages in a different way than the intended meaning of the illustrations developed by adults. These findings are important for the improvement of CCHD and underline the utmost importance of including children in research on health promotion. This study supports the view that 4-year-old children can take an active role in their health and are capable of making health information meaningful.

## INTRODUCTION

Promoting young children’s health is an investment for the future. Ensuring good nutrition, sufficient physical activity, healthy teeth and quality sleep in young children through health promotion will improve their physical and mental health and wellbeing (WHO, 2018). Health promotion is a process enabling people to gain control over the determinants of their health, thereby improving not only health but also wellbeing and quality of life (WHO, 1986). Nurses throughout the world play an important role in promoting health. Some engage in health promotion activities seeking to promote health in communities through political and community driven processes, while most are involved in health education providing individuals with health-related information (Whitehead, 2004; Kemppainen *et al.*, 2013). In the field of promoting children’s health in early childhood, which is a critical time for establishing healthy lifestyle (Waters *et al.*, 2011), health education activities, e.g. in the USA, Australia and the UK mostly focus on risk factors, motivating the child and the caregivers that are particularly at risk or already affected by overweight into a process of behavioural change (Whitehead, 2004; DH, 2009; Lindström and Eriksson, 2011). Nurses in the Swedish Child Health Services (CHS), however, are trained to adapt a salutogenic approach, discussing the positive factors that influence the health of the child and rather supporting the resources and the health knowledge the families already possess (NBHW, 2014). The Swedish CHS are free of charge, and attended by nearly all families with young children, irrespective of social position or ethnicity (NBHW, 2014). Children aged 0–6 years and their families are entitled to a universal health programme including regular health dialogues, health examinations, immunizations and parental support and extra health visits according to need. 

According to the United Nations Convention on the Rights of the Child (UNCRC), children have the right to health and thereby the right to active participation in health promotion activities, but today there is a lack of evidence-based child-centred health promotion models that can be used in CHS (NBHW, 2014, 2018). To fill this gap an intervention that aims to promote a healthy lifestyle was developed and tested for feasibility (Derwig *et al.*, 2019). The Child-Centred Health Dialogue (CCHD) is a structured health dialogue directed to all 4-year-olds visiting CHS, which is conducted in the presence of their caregivers. CCHD is based on the concepts of Child-Centred Care (CCC), health literacy, and empowerment. In CCC the child is seen in the context of its family as well as an active participant in its own health care and its own right (Coyne *et al.*, 2016). Health literacy, which is preferably addressed from an early age, comprises the knowledge, understanding and the capacity to make healthy choices (Velardo and Drummond, 2017). Health literacy can be classified in three dimensions: *functional health literacy* to access and understand health messages in everyday situations, *interactive health literacy* to evaluate and derive meaning, and *critical health literacy* to be able to use and critically analyse health information (Nutbeam, 2000). Empowerment is defined as the social and reflective process in which children and their caregivers become conscious of their knowledge and understanding, their enhanced self-esteem and the development of various social and health skills (Poskiparta *et al.*, 2001). To increase a person’s health literacy and empowerment, health messages should be communicated in ways that support interaction, dialogue and participation, defining participation as active involvement in a situation knowing that one’s actions are acknowledged and may be acted upon (Nutbeam, 2000; Poskiparta *et al.* 2001).

**Fig. 1:**
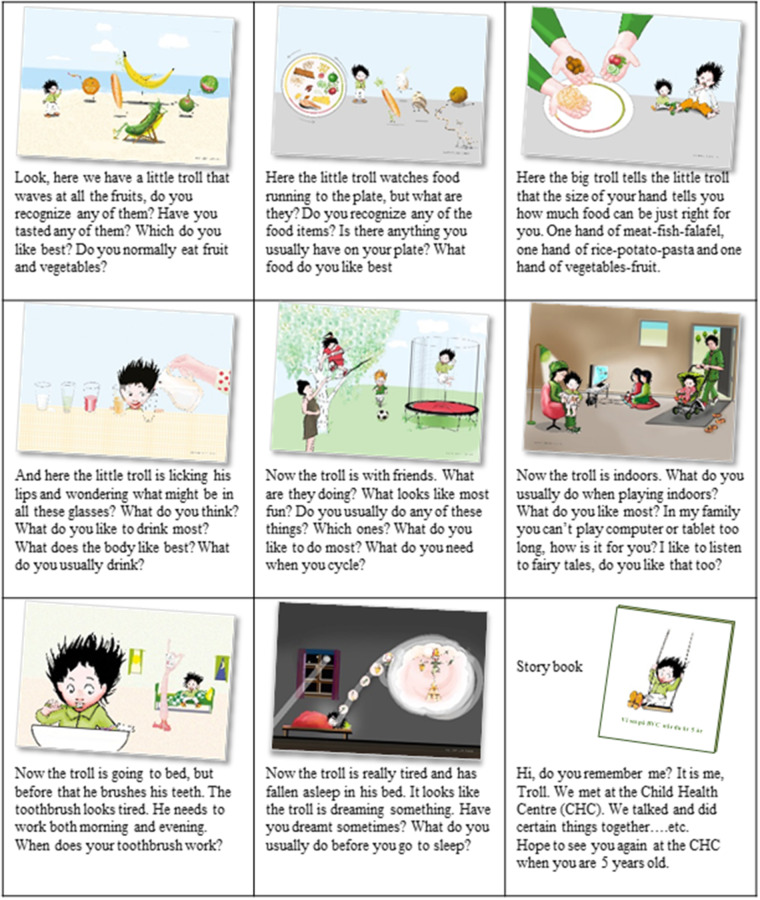
Illustrations used in CCHD with suggested questions. After each illustration, the nurse reflects on the answers, summarizes and empowers the child and the family (Carin Carlsson, illustrator).

CCHD uses eight illustrations based on the most important health messages, according to the best available evidence, promoting good nutrition, sufficient physical activity, healthy teeth and quality sleep (Figure 1; Derwig *et al.*, 2019).

The illustrations form a story about the everyday activities of a troll that meets his ‘fruit and vegetable friends’, eats and drinks, plays, brushes his teeth and goes to bed. Methods that elicit the child’s perspective and promote participation include active listening and discussion followed by reflection (Kangas, 2016). Studies show that if an adult encodes a health message around a story supported by illustrations, young children’s understanding of health information can be improved and in this way support their health literacy (Freeman, 2015; Stålberg, 2017). This approach of participatory learning can give children the ability to control and influence the social environment, which supports the development of both cognitive and social skills (Kangas, 2016). As the child is seen as part of a family, the caregivers are encouraged to participate actively in the health dialogue and at the end of the visit, the child receives a storybook including all the illustrations to read at home. Parental involvement and parental attitudes towards a healthy lifestyle are important determinants for children’s health-related behaviours, and to create a healthy lifestyle, the entire family environment needs to be supported (Waters *et al.*, 2011). Guiding caregivers can be challenging as not only individual factors but also social and structural factors influence health and health-related behaviours (Marmot and Bell, 2012). Previous research shows that the illustrations used in CCHD helped the nurses tailor the information to the needs of individual families resulting in a more neutral relationship and increasing the opportunity to achieve empowerment (Brattwall-Eilert and Tysk, 2018). Also, caregivers felt that the nurses focussed on the family’s strengths and abilities, taking their situation, their own and their child’s descriptions, questions and thoughts into account (Håkansson *et al.*, 2019).

In line with the UNCRC and the Swedish healthcare legislation (2014:821) stating that children have the right to express their views on how they experience matters that concern them, this study aims to explore children’s experiences of CCHD at the 4-year health visit to CHS and capture the child’s perspective.

## METHODS

### Study design

Since knowledge of the phenomenon is scarce, a qualitative design with an inductive approach was chosen as the study design. Both non-participant observations and interviews were used to achieve a better understanding of the phenomenon than using one approach alone.

### Setting and participants

The study was carried out in the southern part of Sweden. Eight nurses responsible for recruiting children were selected. To achieve optimum variation within the sample the nurses were selected purposely based on working experience (<5; 5–10 and >10 years), the location and the Care Need Index (CNI) of the Child Health Centre (CHC) (Elo *et al.*, 2014). A CNI over one implies a higher need for care based on socio-economic factors and is calculated for each CHC based on the enrolled patient population (SCB, 2009). The selected nurses worked at six different CHCs, three of them situated in a larger city and three in smaller communities. Inclusion criteria were 4-year-old children with sufficient speaking skills in Swedish or English that were scheduled for their 4-year health visit during a 2-month period in spring 2018. A strategic sample was drawn with regard to sex, socio-economic background and ethnicity and thirty children and their caregivers were invited to the study by means of an information letter sent by post 1 month prior to the visit. In the end, 21 children participated in the study: 16 children and their caregivers consented to participate in both observation and interview, and 5 children in observation only. The distribution among the nurses was as follows: one nurse recruited one child, four nurses two, two nurses three and one nurse six children. The profile of the children is described in Table 1.

**Table 1:** Profile of the participants

21 children with a mean age of 4 years. Twelve girls and nine boys participated in observations (O1–O21). Nine girls and seven boys participated in interviews (1–16). One observation and one interview was in English. Three children had caregivers born in non-European countries. Six children were accompanied by their father, 12 children by their mother, 3 children by both caregivers during the observation. Six children were joined by siblings. Eight children visited a CHC with high CNI (1.5–0.9) 13 children with low CNI (0.5–0.7). Ten children watched all eight illustrations, two children seven, four children six, three children five and two children four illustrations depending on the context, time frame and child’s interests.

### Data collection

Non-participant observations were used to gain a direct understanding of the phenomenon in its natural context (Liu and Maitlis, 2010) and individual interviews were held to obtain a broad description of the phenomenon (Elo and Kyngäs, 2008).

The first author was present at the 4-year health visit as a non-participant in order to observe CCHD, which lasted on average 10.5 min (range 7.5–14.5 min) of the total 60-min visit. In non-participant observations the researcher takes a passive role and only observes the ongoing verbal and non-verbal interactions between the child, nurse and caregivers, while fully aware of the researcher’s presence (Greig and Taylor, 2001). Key words were written down during the observation and detailed field notes were written directly after each visit to record what had been observed (Liu and Maitlis, 2010).

The interviews were scheduled 0–7 days after the visit, with an average of 3 days and were performed by the first author in the child’s home in the presence of a caregiver to allow the child to feel more secure. A thematic interview guide with open-ended questions was used, focussing on the child’s experiences of the dialogue, reflections on the illustrations and ideas about what the child itself liked to eat, drink and do and what they thought the body in general liked. The guide was used in a flexible way depending on the experiences and illustrations used during CCHD, the context, time frame and child’s interests (Nilsson *et al.*, 2015). The interviews started with a game called Memory in order to get acquainted and build trust and to develop a relationship with the child based on respect and understanding (Doverborg and Pramling Samuelsson, 2012). The game included parts of the CCHD-illustrations and served as a starting point for the children to tell about their experiences by asking: ‘What is it you see, have you seen it before, can you tell me about it?’ Additionally, a troll-puppet was used, asking: ‘Hi, do you remember me, it’s me the Troll. I was present on some pictures with fruits and activities, can you tell me about them?’ The CCHD-illustrations and the storybook were other tools facilitating questions such as ‘What is best for the body, what does it like?’ The first two interviews were intended as pre-interviews to see if the questions were suitable for obtaining rich data. As these did not result in any changes to the interview guide, they were included in the data material. The interviews lasted 36 min on average (range 11–50 min) and were recorded and transcribed verbally by the first author.

### Data analysis

Inductive content analysis was used to analyse the data (Elo and Kyngäs, 2008). First, all authors read the field notes and interviews independently to obtain a sense of the content in its entirety and to identify essential features. In the next step, the first author coded the text by hand, identifying key words or phrases that appeared to elucidate the children’s experiences from their point of view. All authors discussed the codes to reach agreement while making sure that the codes truly described the child’s perspective, which is a delicate process (Stålberg, 2017). The third step was used to compare the codes with regard to similarities and differences and then grouped them into preliminary categories. By going back and forth to the transcribed data, sub-categories and links between them were identified. In the next step the experiences were abstracted into three generic categories describing the various perspectives identified. Table 2 shows this process for the third generic category. At the end of the analysis the preliminary results were discussed with fellow researchers in the field of child health and child participation and one overall main category was formulated (Table 3).

**Table 2:** Children’s experiences of CCHD, third generic category

Units of analysis (participant number)	Codes	Sub-categories	Generic category
The orange is happy, the cucumber is not very happy and not angry (14)	Tell what they see	Interpret the pictures and observe details	Interpret into an understandable story
Everybody is running, except for the troll (13)The lamp is on otherwise you can’t see (1)	Observe the details		
She showed me her hand (*Do you know what she told you?*) I don’t know (10)	Tell what they remember		
The troll likes water best (8)			
He will fall down and mum is helping him, she says : ‘you have to go there’, yes, she says: ‘stop’, so that he will not fall, ‘you should not be up there, you can play if you like’ (12)	See the troll as part of a family	Create a story	
And there you are jumping on the trampoline and there you sit with a few apples and there you drink and there you are brushing your teeth and there you are with all those things and there you are eating fruit and there you sleep (11)	See the pictures as a story		
If you eat a little bit of beans and cucumber and bread and cheese then it will be tasty (7).	Tell what they understand	Create understandable meaning	
You have to brush your teeth… in the night (pointing at the moon) and in the morning (pointing at the sun) (4)			
This one has three hands (15)	Tell what they do not understand		
The troll said: ‘you could eat food with your hands’ (3)			
But there are ice cubes in it, big ice cubes ….I did not drink it (the sugar), most of it was left in it, most of it was left in the soda can (16)			

**Table 3:** Sub-categories, generic categories and main category

Sub-categories	Generic categories	Main category
Enjoy the pictures Participate actively Do what they think is expected Set their limits	Enjoy participating and influencing in an active and social way	Participate as social actors while guided to express their views based upon their own understanding
Tell about their daily life Communicate their preferences Show their knowledge and skills	Express their views based on their daily life	
Interpret the pictures and observe details Create a story Create understandable meaning	Interpret into an understandable story	

### Ethics

Ethical approval was obtained from the Regional Research Ethics Committee (2018/151). The general purpose of research that involves children is to obtain and produce scientific information without harming them. An information letter informed caregivers about the purpose of the study and the data collection. To inform the child, an age-appropriate information letter with illustrations including the troll was included. Informed consent was obtained from both caregivers, who were assured that they were free to withdraw from the study at any time without affecting their or their child’s healthcare in anyway (Beauchamp and Childress, 2013). Oral assent for the observation was obtained from the child just before the 4-year health visit to the CHC, using a puppet resembling the CCHD troll. Oral assent for the interview was requested at the end of the visit, after the consent of the parent, again directly before, and at least once more during the interview at home, notifying that they could end at any time (Beauchamp and Childress, 2013). As children have a tendency to focus on the here-and-now, consent to participate in research needs to be repeated many times so that they can gain a greater understanding (Nilsson *et al.*, 2015). During the research process the rights of the child were constantly guarded and especially during the observation and interview the first author, a paediatrician and medical anthropologist experienced in interviewing children, was attentive to non-verbal signs that the child wanted to stop.

## RESULTS

An overarching main category based on the content of the three generic categories emerged—‘Participate as social actors while guided to express their views based upon their own understanding’, capturing the experiences of CCHD at the 4-year health visit in CHS. The first part—‘participate as social actors’—showed that children liked to participate actively but could influence their choice to participate. The second part—‘while guided to express their views based upon their own understanding’—illustrated that the children liked to express their views based on their daily life but wanted to understand the meaning of the information with which they interacted (Table 3).

### Enjoy participating and influencing in an active and social way

The children showed that they enjoyed the illustrations used. They were curious to see the illustrations and eager to see the next one. When exploring them they scanned the entire illustration while bending over and looking carefully. They appeared interested and listened carefully to what was said by the nurse. Many children laughed when they saw the illustrations and made joyful remarks.

And I like the onion and the cucumber, he is sleeping and snoring and he has slippers (laughing aloud and pointing at the onion). (6)

One child sang the names of all the fruits when she was watching. Even when they did not recognize certain food items, most of the time they showed that they enjoyed the illustrations. The children said that they liked the troll and that they did not think the troll was scary.

The children participated actively in CCHD. They took turns in the dialogue and asked questions such as ‘What is that?’, but also questions like ‘Why can one not sit still and watch television?’ They showed, both non-verbally and verbally, that they wanted to participate and wanted attention to be focussed back on them when the nurse was talking to the caregiver. They moved their body, pointed at the illustrations, asked or said something related to the subject of the conversation between the caregiver and the nurse. The children involved the caregiver or the nurse in the dialogue by exclaiming: ‘Look, look’ or by reminding the nurse what else there was to see: ‘You forgot that one!’

During CCHD the children seemed to read the social situation and immediately acted upon what they thought was expected of them. When they saw the first illustration, most of the children directly started to point and name all the fruits they saw without any instructions: ‘This is a banana, this is a watermelon …’ The children tried to answer the questions asked and were happy to be able to count the glasses or know the colours of the fruits. They sometimes made up names such as ‘watermelon lemonade’ to show that they knew the answer.

Sometimes children changed their answer and responded instead in the way that they thought was expected. For example, when a child said that the troll liked to buy ‘candies’ he changed it to ‘food’ or when they first answered that they liked ‘orange juice’ or ‘lemonade’ best, they corrected it to ‘water’

Nurse: ‘What do you normally drink when you eat dinner?’ Child: ‘Lemonade (pointing at the red coloured glass)’. Nurse: ‘Do you normally drink lemonade when you eat dinner?’ Child: ‘Yes’ (somewhat boldly). Nurse: ‘Mmm’ (looks at the child, waiting for an answer). Child: ‘Water’ (looking at her mum). (O20)

The children influenced the situations during CCHD and set their limits by choosing when they wanted to participate in CCHD and when they did not want to participate any longer. They showed it non-verbally by standing up and turning away from the dialogue or verbally by changing the subject. Other children chose not to answer and said something else like ‘It slipped my mind’ or ‘I think I told you a lot’. Another strategy was to suggest other things to do. Two children interrupted the dialogue because they wanted to show their brushes to the nurse.

### Express their views based on their daily life

The children talked about their daily lives. They spoke about the things they normally ate and drank at home or at day care, but also what they drank or ate on special occasions, such as what they drank or ate as a treat, when they visited their grandparents or during the weekend or holidays. Most of the children said that they drank water and milk normally and lemonade, chocolate milk and juice on special occasions. They also communicated, without being asked for it, what they were not allowed to eat every day, such as pancakes, fast food, candies or soft drinks, because they would eat too much and get tummy ache or their teeth would go bad. Some children told about their food allergy or intolerance. They described their preferences and said what they liked and were happy to tell about their activities in daily life. They mentioned which food and activities they liked and did not like, especially those related to what was shown on the illustrations. In their stories they included activities at home, at day care, with friends and with caregivers and siblings.

They also communicated their knowledge and skills and revealed what the body liked and needed. According to the children the body liked fruit and vegetables but also ordinary food, such as pasta, peas and meatballs. They said that fruit and vegetables were healthy and described them as elements the body needed. They said that the body would not live without eating them and that the body would feel good and become big and strong when eating them.

I think that it is good to eat fruit and vegetables because…then you can become as strong as the Hulk (lifts his arms like a body builder). (11)

They said that the body and teeth liked water best and that drinking water was something you had to do. But even so, they sometimes stated that juice and milk were better than water. The children proudly communicated their ability to brush their own teeth and pointed out that you had to use toothpaste. The children also knew that if you did not have a safety net around the trampoline you would fall and that you had to wear a helmet when you cycled.

### Interpret into an understandable story

When the children looked at the illustrations they talked about what they saw. They saw happy fruit waving, swimming, dancing, smiling and talking (Table 2).

It is a little boy that wants to meet them. The boy wants to eat the orange, but the orange has a mouth, he is really happy. (O9)

The children described the details they observed, such as ‘that (banana) looks like a bird, it has wings’ or ‘the troll is outside, because he is wearing a green jacket’. They saw glasses of the same size, one with bubbles and one that was empty, and observed the trampoline with a safety net but no steps and the troll reading without shoes.

They remembered the troll greeting fruit and vegetables and talking about drinking milk and liking water best. They recalled an illustration showing a plate with peas, cucumber and tomato and that the nurse took their hand or showed her own hand, but they could not recollect what the nurse had said. Additionally, the children remembered the troll jumping on the trampoline, a girl climbing the tree, the troll brushing his teeth in the morning and at night and the troll dreaming.

Moreover, the children saw the different illustrations as an ongoing story and described the troll as part of a family. They distinguished a big brother, father, mother, baby in the stroller, sister with a pony tail in the tree, sisters in front of the television and grandmother reading a book. ‘The mother with a baby in her belly helped the sister climb the tree’ but ‘warned her not to climb too high because it was dangerous to climb that high’. They referred to their own families and to their own homes. ‘They are sisters, I have two sisters as well’ and they are sitting ‘on the floor and not on the couch like at home’.

During the observations and interviews the children communicated what they understood but also spoke about the messages they did not understand. They recognized, e.g. that fruit and vegetables were good to eat and designated them as ‘real food’. They also understood that the plate with all the different kinds of food items implied that you should eat ‘various things’ and a ‘little bit of everything' to make it ‘tasty’.

The illustration designed to demonstrate portion size was perceived as ambiguous by the children. On the one hand the children recognized the troll together with a bigger troll and understood that the three hands with different types of food explicated that you should eat one of each: one hand with spaghetti, one with meatballs and one with vegetables. On the other hand, they did not understand the meaning of the three hands and were wondering why there was a person with three hands.

Why does he have three arms? (O4)

They likewise did not understand why the hands contained food. They stated that you were not allowed to eat with your hands and should eat with cutlery or should have food on your plate instead. Some children thought about it a bit longer and concluded that some vegetables and fruits could still be eaten with your hands but not meatballs and spaghetti, because that would become ‘messy’. In the children’s experience the troll communicated ‘that you could eat food with your hands’.

The illustration with the drinks also contained several different meanings that did not fit well together. The children understood that the troll liked water best and that ‘the jug should contain water’, but they also indicated that the troll liked ‘all’ drinks.

You should drink milk and soft drink and lemonade and orange juice. He tells us those are good. (12)

When the nurses explained that lemonade, juice and soft drinks contained a lot of sugar, not all children understood what that meant. One child could figure that her glass of soda contained ‘ice cubes’, but she had not been able to observe sugar in her drink and suggested that the sugar must have been left behind in the can.

The illustrations showing activity and sedentary behaviour were well understood. The children distinguished clear differences between the illustration where ‘all were active’ and the illustration where ‘all were sitting still’. They also seemed to understand the illustration with the troll brushing his teeth well and stated that the troll said that you had to brush your teeth with toothpaste in the morning and in the evening. They pointed out the sun and the moon as distinct signs for morning and evening and noted that the troll had ‘squeezed toothpaste out of the tube’. They also understood that the troll was dreaming in the last illustration.

## DISCUSSION

This study elucidates the experiences of 4-year-old children participating in CCHD in the Swedish CHS. The children participated as social actors guided to express their views based upon their own understanding. The study confirms that children like to be actively involved in health care situations (Coyne *et al.*, 2016; Stålberg, 2017). The children set their limits when they did not want to participate any longer and could in this way influence their choice to participate. Being able to choose whether or not to participate and being shown trust and respect empowers children and is positive for their health and wellbeing (Kostenius, 2008). As described by others, children have their own unique social worlds, experiences and understandings and are able to be active and reflective in interpreting health messages (Fairbrother *et al.*, 2016; Wiseman *et al.*, 2018). The children seemed to enjoy the interactive dialogue which they could influence and in which they could participate in an active and social way. They were able to read the social situation and did what they thought was expected. When changing their answer from lemonade to water they demonstrated their understanding of the dominant health message in the Swedish context that drinking water is healthy. This finding illustrates that children are social agents who construct understandings reflecting their experiences from daily life, the context and the key societal values in which they live (Lanigan, 2011; Freeman, 2015; Wiseman *et al.*, 2018). In line with previous research, the children were health-conscious and recognized basic health concepts, supporting the view that children from a young age can take an active role in their health (Bröder *et al.*, 2017; Velardo and Drummond, 2017). They understood the dichotomy that certain food items could be consumed every day, in contrast to those that could only be enjoyed on special occasions such as pancakes, fast food, candies or soft drinks. Children as young as 3 years understand the relationship between health and eating and can classify food as healthy and unhealthy (Schultz and Danford, 2016). In this study, the 4-year-old children were aware that eating fruit and vegetables and drinking water were healthy choices and that drinking water was best for your teeth. Even children with food allergy or intolerance had an active role in their choices of favourite foods and rejected food that they did not tolerate.

The study also showed that CCHD enabled the children to express their views based on their daily life. By questioning, actively listening, responding and following the child’s initiatives, the nurses supported the children to voice their preferences, knowledge and skills and permitted to process the health messages from the child’s perspective. Children, irrespective of their stage of cognitive and psychological development, want to be guided by attentive adults in encoding health messages and want to reflect upon them in order to make the information meaningful (Sommer *et al.*, 2009). Through these participatory strategies and by passing on empowering experiences, children become more aware of the health issues facing them and may take action to maintain and improve their health (Borzekowski, 2009). The children reflected on the usefulness of health messages and mobilized their personal, embodied experiences to filter health information in a manner that was meaningful for them (Freeman, 2015; Fairbrother *et al.*, 2016). ‘Brushing your teeth twice a day with toothpaste’ and ‘having a safety net around the trampoline’ or ‘wearing a helmet when cycling’ were well-embedded health messages for the children participating in this study.

An important finding was that the children interpreted the illustrations into an understandable story, implying that they had the ability to access and understand information, as described in the first two of Nutbeam’s (Nutbeam, 2000) dimensions of health literacy. The children did not passively absorb the health messages but rather worked with them to create meaning (Fairbrother *et al.*, 2016), as demonstrated by their experiences of the illustration of portion size. The interviews revealed that none of the children remembered the health message, that the size of your hand helps you to estimate how much food you can eat as the children presumably were captivated by *the person with three hands* and by the fact that this person had food in his hands. The children understood the illustration but in a different way. Although the nurses explicitly told the children that the illustration did not mean that food should be eaten with your hands, the children interpreted it in that way and questioned why there was a person with three hands.

These findings show that children are capable of assessing the relevance of information to their own situation as in critical health literacy (Nutbeam, 2000). The children’s experiences differ from the experiences of the nurses executing CCHD, who described the illustration of portion size as their favourite and thought the three hands clearly illustrated how much a child needed to eat and increased understanding for both children and caregivers (Brattwall-Eilert and Tysk, 2018). Caregivers likewise appreciated this specific illustration as it gave them a better understanding of portion size, especially when they were worried about their child not eating enough (Håkansson *et al.*, 2019).

The illustration that shows the troll with different kinds of beverages—intended to open up a dialogue about sugary drinks and to promote the health message that water is best—was also experienced in a surprising way. The children were aware of the fact that water is best for both body and teeth but stated that the illustration showed that the troll liked *all* drinks. Besides, some children had difficulties imagining that certain beverages contained sugar as they literally did not *see* sugar in their drinks.

In this way the present study reveals that 4-year-old children, given the opportunity to speak for themselves, elucidating the child’s perspective, interpreted the health messages in a different way than the intended meaning of the illustrations developed by adults having a child perspective. In order to make health information meaningful to children the child’s perspective and not only the child perspective should always be included in the development of child-centred interventions.

It is important to emphasize that CCHD recognizes that children belong to a family and that their caregivers play a key role in shaping a healthy lifestyle for their children. Håkansson described how caregivers felt supported and confirmed in promoting good food and eating habits, became more responsive to their children’s appetite and were impressed by the child’s knowledge and understanding expressed during CCHD (Håkansson *et al.*, 2019). In CCHD nurses need to be attentive to both the children and their caregivers. They should use their social and pedagogical skills to support the reflective process in CCHD that enables all participants to become conscious of their knowledge and understanding of health and in this way strengthen their health literacy and empowerment.

## METHODOLOGICAL CONSIDERATIONS

To facilitate trustworthiness a clear description of the context, participants, data collection and process of analysis is given (Elo *et al.*, 2014). The children involved were of different gender, came from families with varied socio-economic status, education and living conditions and were recruited by nurses with varying work experience at six CHCs in different locations. Some children were more talkative and others more taciturn. One child did not talk at all but communicated with gestures and by making small drawings. Parental factors such as lifestyle factors and body composition were not taken into account as the aim of the present study was to elucidate the experiences of children that participated in CCHD and not to investigate how parental factors influence children’s experiences nor what actual knowledge the children achieved. Data triangulation of observations and interviews ensures trustworthy data collection and integrates insights from both verbal and non-verbal experiences, which is a reliable way to visualize 4-year-old children’s views in order to build a deeper understanding (Flick, 2007; Krippendorff, 2013). As this study endeavoured to capture the child’s perspective, the researchers had to be attentive to explore and describe the data from the child’s point of view during the entire research process (Söderbäck *et al.*, 2011). It is unavoidable, however, that part of the experiences expressed by the children have been interpreted from an adult point of view as the researchers were adults (Stålberg, 2017). A limitation of the study is that children without Swedish or English-speaking skills were excluded and that only three children had a non-European background. As for transferability, it is important to recognize that the study was carried out in the southern county of Sweden, where society and in particular CHS might be organized differently from other countries and that only a relatively small number of children participated. However, the findings might be transferable to other health promotion settings as several findings are supported by international research and a larger sample might not have revealed additional information (Guest *et al.*, 2006).

## CONCLUSION

The study indicates that CCHD with its child-centred approach and use of illustrations created a health dialogue in which children enjoyed participating and were able to express their views. In this way, 4-year-old children interacted with health information and constructed health-relevant understandings in the context of their everyday life. This study supports the view that 4-year-old children can take an active role in their health and are capable of making health information meaningful. This is important for both health professionals working in CHS and children’s caregivers, and it is in line with the UNCRC, incorporated in Swedish law since January 2020 (2018:1197).

This study also shows how vital it is to include children in research on health promotion, as health promotion activities for families with preschool children are provided worldwide. The findings of this study are the starting point for a revision of the illustrations in cooperation with 4-year-olds from various socio-economic backgrounds and the illustrator. CCHD is moreover tested in an RCT for its effectiveness and cost effectiveness and compares parental self-efficacy and feeding practices in families that received either CCHD or usual care. Future research could also include a follow-up of the children concerning their achieved knowledge after CCHD, in both the short and the long term. An assessment of how nurses are able to ensure both the child and the child’s perspective in CCHD and what strategies they use when meeting families with other views of health and health-related behaviours than the ones present in CCHD are other questions to investigate. These findings could provide a better understanding of CCHD and are important to consider before implementing CCHD in the Swedish Child Health Programme.

## ETHICAL APPROVAL

Ethical approval was obtained from the Regional Research Ethics Committee (2018/151).
